# Evaluation of Abbott BinaxNOW Rapid Antigen Test for SARS-CoV-2 Infection at Two Community-Based Testing Sites — Pima County, Arizona, November 3–17, 2020

**DOI:** 10.15585/mmwr.mm7003e3

**Published:** 2021-01-22

**Authors:** Jessica L. Prince-Guerra, Olivia Almendares, Leisha D. Nolen, Jayleen K. L. Gunn, Ariella P. Dale, Sean A. Buono, Molly Deutsch-Feldman, Suganthi Suppiah, LiJuan Hao, Yan Zeng, Valerie A. Stevens, Kristen Knipe, Justine Pompey, Christine Atherstone, David P. Bui, Tracy Powell, Azaibi Tamin, Jennifer L. Harcourt, Patricia L. Shewmaker, Magdalena Medrzycki, Phili Wong, Shilpi Jain, Alexandra Tejada-Strop, Shannon Rogers, Brian Emery, Houping Wang, Marla Petway, Caitlin Bohannon, Jennifer M. Folster, Adam MacNeil, Reynolds Salerno, Wendi Kuhnert-Tallman, Jacqueline E. Tate, Natalie J. Thornburg, Hannah L. Kirking, Khalilullah Sheiban, Julie Kudrna, Theresa Cullen, Kenneth K. Komatsu, Julie M. Villanueva, Dale A. Rose, John C. Neatherlin, Mark Anderson, Paul A. Rota, Margaret A. Honein, William A. Bower

**Affiliations:** ^1^CDC COVID-19 Response Team; ^2^Arizona Department of Health Services; ^3^Epidemic Intelligence Service, CDC; ^4^Pima County Health Department, Tucson, Arizona.

*On January 19, 2021, this report was posted online as an *MMWR *Early Release.*

Rapid antigen tests, such as the Abbott BinaxNOW COVID-19 Ag Card (BinaxNOW), offer results more rapidly (approximately 15–30 minutes) and at a lower cost than do highly sensitive nucleic acid amplification tests (NAATs) (*1*). Rapid antigen tests have received Food and Drug Administration (FDA) Emergency Use Authorization (EUA) for use in symptomatic persons (*2*), but data are lacking on test performance in asymptomatic persons to inform expanded screening testing to rapidly identify and isolate infected persons (*3*). To evaluate the performance of the BinaxNOW rapid antigen test, it was used along with real-time reverse transcription–polymerase chain reaction (RT-PCR) testing to analyze 3,419 paired specimens collected from persons aged ≥10 years at two community testing sites in Pima County, Arizona, during November 3–17, 2020. Viral culture was performed on 274 of 303 residual real-time RT-PCR specimens with positive results by either test (29 were not available for culture). Compared with real-time RT-PCR testing, the BinaxNOW antigen test had a sensitivity of 64.2% for specimens from symptomatic persons and 35.8% for specimens from asymptomatic persons, with near 100% specificity in specimens from both groups. Virus was cultured from 96 of 274 (35.0%) specimens, including 85 (57.8%) of 147 with concordant antigen and real-time RT-PCR positive results, 11 (8.9%) of 124 with false-negative antigen test results, and none of three with false-positive antigen test results. Among specimens positive for viral culture, sensitivity was 92.6% for symptomatic and 78.6% for asymptomatic individuals. When the pretest probability for receiving positive test results for SARS-CoV-2 is elevated (e.g., in symptomatic persons or in persons with a known COVID-19 exposure), a negative antigen test result should be confirmed by NAAT (*1*). Despite a lower sensitivity to detect infection, rapid antigen tests can be an important tool for screening because of their quick turnaround time, lower costs and resource needs, high specificity, and high positive predictive value (PPV) in settings of high pretest probability. The faster turnaround time of the antigen test can help limit transmission by more rapidly identifying infectious persons for isolation, particularly when used as a component of serial testing strategies.

Paired upper respiratory swabs were collected at the same timepoint from persons aged ≥10 years receiving testing for SARS-CoV-2, the virus that causes coronavirus disease 2019 (COVID-19), at two Pima County Health Department community testing sites during November 3–17 (site A) and November 8–16 (site B). The sites offered SARS-CoV-2 testing to anyone in the community who wanted testing. A questionnaire capturing demographic information and current and past–14-day symptoms was administered to all participants. At both sites, a health care professional first collected a bilateral anterior nasal swab, using a swab provided in the BinaxNOW kit, immediately followed by a bilateral nasopharyngeal (NP) swab for real-time RT-PCR testing. Anterior nasal swabs were immediately tested on-site using the BinaxNOW antigen test according to the manufacturer's instructions (*4*). NP swabs were stored in phosphate buffered saline at 39°F (4°C) and analyzed within 24–48 hours by real-time RT-PCR using either the CDC 2019-nCoV Real-Time RT-PCR Diagnostic Panel for detection of SARS-CoV-2 (*5*) (2,582 swabs) or the Fosun COVID-19 RT-PCR Detection Kit (*6*) (837 swabs). Viral culture*^,^^†^ was attempted on 274 of 303 residual real-time RT-PCR specimens if either the real-time RT-PCR or BinaxNOW antigen test result was positive (the remaining 29 were not available for viral culture). Results from real-time RT-PCR and the BinaxNOW antigen test were compared to evaluate sensitivity, specificity, negative predictive value (NPV), and PPV. Statistical analyses were performed using SAS (version 9.4; SAS Institute). Cycle threshold (Ct) values from real-time RT-PCR were compared using a Mann-Whitney U Test; 95% confidence intervals (CIs) were calculated using the exact binomial method. The investigation protocol was reviewed by CDC and determined to be nonresearch and was conducted consistent with applicable federal law and CDC policy.^§^

Paired upper respiratory swabs were collected from 3,419 persons, including 1,458 (42.6%) from site A and 1,961 (57.4%) from site B (Table 1). Participants ranged in age from 10 to 95 years (median = 41 years) with 236 (6.9%) aged 10–17 years, 1,885 (55.1%) aged 18–49 years, 743 (21.7%) aged 50–64 years, and 555 (16.2%) aged ≥65 years. Approximately one third (31.4%) of participants identified as Hispanic or Latino, and three quarters (75.1%) identified as White.

**TABLE 1:** Characteristics of persons providing paired upper respiratory swabs (N = 3,419)* for the Abbott BinaxNOW COVID-19 Ag Card Point of Care Diagnostic Test and real-time reverse transcription–polymerase chain reation (RT-PCR) testing^†^ for SARS-CoV-2 at two community-based testing sites, by test results — Pima County, Arizona, November 2020

Characteristic	Total no. of persons (column %)	No. of persons (row %)^§^			
		Antigen-positive	Real-time RT-PCR–positive	Real-time RT-PCR–positive, antigen-negative	Real-time RT-PCR–negative, antigen-positive
**Total**	**3,419 (100)**	**161 (4.7)**	**299 (8.7)**	**142 (4.2)**	**4 (0.1)**
**Testing site**					
A	**1,458 (42.6)**	72 (4.9)	145 (9.9)	74 (5.1)	1 (0.1)
B	**1,961 (57.4)**	89 (4.5)	154 (7.9)	68 (3.5)	3 (0.2)
**Sex**					
Male	**1,290 (37.7)**	74 (5.7)	138 (10.7)	65 (5.0)	1 (0.1)
Female	**1,681 (49.2)**	76 (4.5)	127 (7.6)	54 (3.2)	3 (0.2)
Undisclosed	**448 (13.1)**	11 (2.5)	34 (7.6)	23 (5.1)	0 (—)
**Ethnicity**					
Hispanic/Latino	**1,075 (31.4)**	86 (8.0)	150 (14.0)	65 (6.0)	1 (0.1)
Not Hispanic or Latino	**1,930 (56.4)**	63 (3.3)	118 (6.1)	58 (3.0)	3 (0.2)
Undisclosed	**414 (12.1)**	12 (2.9)	31 (7.5)	19 (4.6)	0 (—)
**Race**					
White	**2,567 (75.1)**	110 (4.3)	204 (7.9)	98 (3.8)	4 (0.2)
Black/African American	**83 (2.4)**	3 (3.6)	8 (9.6)	5 (6.0)	0 (—)
American Indian/Alaska Native	**69 (2.0)**	1 (1.4)	2 (2.9)	1 (1.4)	0 (—)
Asian	**84 (2.5)**	4 (4.8)	10 (11.9)	6 (7.1)	0 (—)
Native Hawaiian/Pacific Islander	**24 (0.7)**	1 (4.2)	1 (4.2)	0 (—)	0 (—)
Undisclosed	**592 (17.3)**	42 (7.1)	74 (12.5)	32 (5.4)	0 (—)
**Age group, yrs**					
10–17	**236 (6.9)**	10 (4.2)	22 (9.3)	13 (5.5)	1 (0.4)
18–49	**1,885 (55.1)**	91 (4.8)	178 (9.4)	89 (4.7)	2 (0.1)
50–64	**743 (21.7)**	41 (5.5)	69 (9.3)	29 (3.9)	1 (0.1)
≥65	**555 (16.2)**	19 (3.4)	30 (5.4)	11 (2.0)	0 (—)
Median age (range)	**41 (10–95)**	40 (13–84)	38 (11–84)	35 (11–83)	27 (16–63)
**Current symptoms^¶^**					
≥1	**827 (24.2)**	113 (13.7)	176 (21.3)	63 (7.6)	0 (—)
None	**2,592 (75.8)**	48 (1.9)	123 (4.7)	79 (3.0)	4 (0.2)
Days from symptom onset**					
Median (range)	**4 (0–210)**	3 (0–14)	4 (0–45)	4 (0–45)	2 (0–12)
0–3	**382 (11.2)**	59 (15.4)	84 (22.0)	25 (6.5)	0 (—)
4–7	**280 (8.2)**	42 (15.0)	58 (20.7)	16 (5.7)	0 (—)
8–10	**43 (1.3)**	6 (14.0)	12 (27.9)	6 (14.0)	0 (—)
11–14	**63 (1.8)**	6 (9.5)	16 (25.4)	10 (15.9)	0 (—)
>14	**55 (1.6)**	0 (—)	6 (10.9)	6 (10.9)	0 (—)
≤7	**662 (19.4)**	101 (15.3)	142 (21.5)	41 (6.2)	0 (—)
**Exposure to a diagnosed COVID-19 case^††^**					
Yes	**1,138 (33.3)**	93 (8.2)	162 (14.2)	71 (6.2)	2 (0.2)
No/Unknown	**2,281 (66.7)**	68 (3.0)	137 (6.0)	71 (3.1)	2 (0.1)
Days since last exposure, median (range)	**5 (0–14)**	4 (0–14)	3 (0–14)	1 (0–14)	9 (4–14)
**Positive test results in past 90 days^§§^**					
Yes	**179 (5.2)**	22 (12.3)	83 (46.4)	62 (34.6)	1 (14.3)
No/Unknown	**3,239 (94.7)**	139 (4.3)	216 (6.7)	80 (2.5)	3 (42.9)

**Abbreviation:** COVID-19 = coronavirus disease 2019.

* Includes 113 persons who received testing multiple times and were included more than once in the analysis.

^†^ Testing with real-time RT-PCR was performed using the CDC 2019-nCoV Real-Time RT-PCR Diagnostic Panel for detection of SARS-CoV-2 (2,582 participants) or Fosun assay (837 participants).

^§^ Only selected categories shown; therefore, row numbers and percentages do not sum to total or 100%.

^¶^ Participants were asked whether they had each individual sign or symptom from a list based on the Council of State and Territorial Epidemiologists’ clinical criteria for COVID-19 interim case definition, which include fever, cough, shortness of breath, fatigue, sore throat, headache, muscle aches, chills, nasal congestion, difficulty breathing, diarrhea, nausea, vomiting, abdominal pain, rigors, loss of taste, and loss of smell (https://cdn.ymaws.com/www.cste.org/resource/resmgr/ps/positionstatement2020/Interim-20-ID-02_COVID-19.pdf).

** Based on one or more symptoms.

^††^ Exposure was defined as close contact (within 6 ft for ≥15 min) in the 14 days before the day of testing with a person with diagnosed COVID-19.

^§§^ Received positive real-time RT-PCR or antigen test result.

At the time of testing, 827 (24.2%) participants reported at least one COVID-19–compatible sign or symptom,^¶^ and 2,592 (75.8%) were asymptomatic. Among symptomatic participants, 113 (13.7%) received a positive BinaxNOW antigen test result, and 176 (21.3%) received a positive real-time RT-PCR test result. Among asymptomatic participants, 48 (1.9%) received a positive BinaxNOW antigen test result, and 123 (4.7%) received a positive real-time RT-PCR test result.

Testing among symptomatic participants indicated the following for the BinaxNOW antigen test (with real-time RT-PCR as the standard): sensitivity, 64.2%; specificity, 100%; PPV, 100%; and NPV, 91.2% (Table 2); among asymptomatic persons, sensitivity was 35.8%; specificity, 99.8%; PPV, 91.7%; and NPV, 96.9%. For participants who were within 7 days of symptom onset, the BinaxNOW antigen test sensitivity was 71.1% (95% CI = 63.0%–78.4%), specificity was 100% (95% CI = 99.3%–100%), PPV was 100% (95% CI = 96.4%–100%), and NPV was 92.7% (95% CI = 90.2%–94.7%). Using real-time RT-PCR as the standard, four false-positive BinaxNOW antigen test results occurred, all among specimens from asymptomatic participants. Among 299 real-time RT-PCR positive results, 142 (47.5%) were false-negative BinaxNOW antigen test results (63 in specimens from symptomatic persons and 79 in specimens from asymptomatic persons).

**TABLE 2:** Test results and performance characteristics of the Abbott BinaxNOW COVID-19 Ag Card Point of Care Diagnostic Test (BinaxNOW antigen test) compared with real-time reverse transcription–polymerase chain reaction (RT-PCR) for testing received among asymptomatic and symptomatic persons at two community-based testing sites — Pima County, Arizona, November 2020

Results and Performance	Real-time RT-PCR, no. of tests		
	Positive	Negative	Total
**BinaxNOW antigen test result**			
**All participants (N = 3,419)**			
Positive	157	4	**161**
Negative	142	3,116	**3,258**
**Total**	**299**	**3,120**	**3,419**
**Symptomatic (≥1 symptom) (n = 827)**			
Positive	113	0	**113**
Negative	63	651	**714**
**Total**	**176**	**651**	**827**
**Asymptomatic (n = 2,592)**			
Positive	44	4	**48**
Negative	79	2,465	**2,544**
**Total**	**123**	**2,469**	**2,592**
**BinaxNOW antigen test performance, % (95% CI)**			
**All participants (N = 3,149)**			
Sensitivity	52.5 (46.7–58.3)		
Specificity	99.9 (99.7–100.0)		
PPV	97.5 (93.8–99.3)		
NPV	95.6 (94.9–96.3)		
**Symptomatic (n = 827)**			
Sensitivity	64.2 (56.7–71.3)		
Specificity	100.0 (99.4–100.0)		
PPV	100.0 (96.8–100.0)		
NPV	91.2 (88.8–93.1)		
**Asymptomatic (n = 2,592)**			
Sensitivity	35.8 (27.3–44.9)		
Specificity	99.8 (99.6–100.0)		
PPV	91.7 (80–7.7)		
NPV	96.9 (96.1–97.5)		

**Abbreviations:** CI = confidence interval; COVID-19 = coronavirus disease 2019; NPV = negative predictive value; PPV = positive predictive value.

Virus was recovered from 96 (35.0%) of 274 analyzed specimens that were positive by either test, including 85 (57.8%) of 147 with concordant positive results and 11 (8.9%) of 124 with false-negative BinaxNOW antigen test results. Virus was not recovered from any of the three available specimens with false-positive BinaxNOW antigen test results. Among the 224 specimens undergoing viral culture that were analyzed with the CDC 2019-nCoV Real-Time RT-PCR Diagnostic Panel for detection of SARS-CoV-2, median Ct values** were significantly higher for specimens with false-negative BinaxNOW antigen test results, indicating lower viral RNA levels than in those with concordant positive results (33.9 versus 22.0 in specimens from symptomatic persons [p<0.001] and 33.9 versus 22.5 in specimens from asymptomatic persons [p<0.001]) (Figure). Median Ct values for SARS-CoV-2 culture-positive specimens (22.1) were significantly lower than were those for culture-negative specimens (32.8) (p<0.001), indicating higher levels of viral RNA in culture-positive specimens. Among specimens with positive viral culture, the sensitivity of the BinaxNOW antigen test compared with real-time RT-PCR in specimens from symptomatic participants was 92.6% (95% CI = 83.7%–97.6%) and in those from asymptomatic participants was 78.6% (95% CI = 59.1%–91.7%).

**FIGURE:**
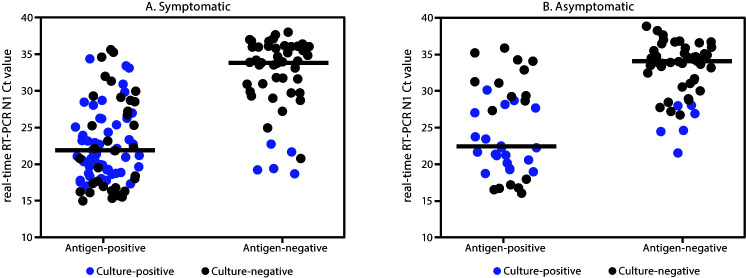
Abbott BinaxNOW COVID-19 Ag Card Point of Care Diagnostic Test (antigen test) results, N1 cycle threshold (Ct) values,* and viral culture results^†^ among A) symptomatic (N = 136)^§^ and B) asymptomatic (N = 88)^¶^ participants receiving positive SARS-CoV-2 real-time reverse transcription–polymerase chain reaction (RT-PCR) test results at two community-based testing sites — Pima County, Arizona, November 2020 * Only those specimens that were analyzed using the CDC 2019-nCoV Real-Time RT-PCR Diagnostic Panel for detection of SARS-CoV-2 and that were analyzed using viral culture are included in the graph. † Twenty specimens with Ct values <18 had positive antigen and real-time RT-PCR results but were culture negative. The culture showed evidence of cytopathic effects and had presence of SARS-CoV-2 RNA as detected by real-time RT-PCR in the first passage culture, but viral recovery was not two Ct values lower than the corresponding clinical specimen Ct. § Antigen test results: 88 positive and 48 negative; median Ct values indicated with black line: 22.0 for antigen-positive specimens and 33.9 for antigen-negative specimens. ¶ Antigen test results: 37 positive and 51 negative; median Ct values indicated with black line: 22.5 for antigen-positive specimens and 33.9 for antigen-negative specimens.

## Discussion

In this evaluation, using real-time RT-PCR as the standard, the sensitivity of the BinaxNOW antigen test was lower among specimens from asymptomatic persons (35.8%) than among specimens from symptomatic persons (64.2%). Specificity (99.8%–100%) was high in specimens from both asymptomatic and symptomatic groups. The prevalence of having SARS-CoV-2 real-time RT-PCR positive test results in this population was moderate (8.7% overall; 4.7% for asymptomatic participants); administering the test in a lower prevalence setting will likely result in a lower PPV.^††^ Among 11 participants with antigen-negative, real-time RT-PCR–positive specimens with positive viral culture, five were symptomatic and six asymptomatic. Some antigen-negative, real-time RT-PCR–positive specimens possibly could represent noninfectious viral particles, but some might also represent infectious virus not detected by the antigen test. In a clinical context, real-time RT-PCR provides the most sensitive assay to detect infection. Viral culture, although more biologically relevant than real-time RT-PCR, is still an artificial system and is subject to limitations. Numerous biological (e.g., individual antibody status and specific sequence of the virus) and environmental (e.g., storage conditions and number of freeze-thaw cycles) variables can affect the sensitivity and outcome of viral culture. Despite the limitations of interpreting culture-negative specimens, a positive viral culture is strong evidence for the presence of infectious virus. The performance of the BinaxNOW antigen test compared with real-time RT-PCR was better for those specimens with positive viral culture than for all specimens, with a sensitivity of 92.6% for specimens from symptomatic persons and 78.6% for those from asymptomatic persons. The results of the current evaluation differ from those of an evaluation of the BinaxNOW antigen test in a community screening setting in San Francisco (*7*), which found a BinaxNOW antigen test overall sensitivity of 89.0% among specimens from all 3,302 participants, regardless of the Ct value of the real-time RT-PCR–positive specimens.

The findings in this investigation are subject to at least five limitations. First, anterior nasal swabs were used for BinaxNOW antigen testing, but NP swabs were used for real-time RT-PCR testing, which might have contributed to increased detection for the real-time RT-PCR assay (*8*). Second, participants might have inadvertently reported common nonspecific symptoms as COVID-19–compatible symptoms. Third, this investigation evaluated the BinaxNOW antigen test, and results presented here cannot be generalized to other FDA-authorized SARS-CoV-2 antigen tests. Fourth, the BinaxNOW antigen test characteristics might be different depending on whether an individual had previously tested positive. Finally, many factors might limit the ability to culture virus from a specimen, and the inability to detect culturable virus should not be interpreted to mean that a person is not infectious.

Public health departments are implementing various strategies to reduce or prevent SARS-CoV-2 transmission, including expanded screening testing for asymptomatic persons (*3*). Because estimates suggest that over 50% of transmission occurs from persons who are presymptomatic or asymptomatic (*9*), expanded screening testing, potentially in serial fashion for reducing transmission in specific venues (e.g., institutions of higher education, schools, and congregate housing settings), is essential to interrupting transmission (*3*).

Rapid antigen tests can be an important tool for screening because of their quick turnaround time, lower requirement for resources, high specificity, and high PPV in settings of high pretest probability (e.g., providing testing to symptomatic persons, to persons with a known COVID-19 exposure, or where community transmission is high). Importantly, the faster time from testing to results reporting can speed isolation of infectious persons and will be particularly important in communities with high levels of transmission.

Although the sensitivity of the BinaxNOW antigen test to detect infection was lower compared with real-time RT-PCR, it was relatively high among specimens with positive viral culture, which might reflect better performance for detecting infection in a person with infectious virus present. Community testing strategies focused on preventing transmission using antigen testing should consider serial testing (e.g., in kindergarten through grade 12 schools, institutions of higher education, or congregate housing settings), which might improve test sensitivity in decting infection (*10*). When the pretest probability for receiving positive SARS-CoV-2 test results is elevated (e.g. for symptomatic persons or for persons with a known COVID-19 exposure) a negative antigen test result should be confirmed by NAAT. Asymptomatic persons who receive a positive BinaxNOW antigen test result in a setting with a high risk for adverse consequences resulting from false-positive results (e.g. in long-term care facilities) should also receive confirmatory testing by NAAT (*1*).

Despite their reduced sensitivity to detect infection compared with real-time RT-PCR, antigen tests might be particularly useful when real-time RT-PCR tests are not readily available or have prolonged turnaround times. Persons who know their positive test result within 15–30 minutes can isolate sooner, and contact tracing can be initiated sooner and be more effective than if a test result is returned days later. Serial antigen testing can improve detection, but consideration should be given to the logistical and personnel resources needed. All persons receiving negative test results (NAAT or antigen) should be counseled that wearing a mask, avoiding close contact with persons outside their household, and washing hands frequently remain critical to preventing the spread of COVID-19.^§§^

**Summary**

**What is already known about this topic?**

The BinaxNOW rapid antigen test received Emergency Use Authorization by the Food and Drug Administration for testing specimens from symptomatic persons; performance among asymptomatic persons is not well characterized.

**What is added by this report?**

Sensitivity of the BinaxNOW antigen test, compared with polymerase chain reaction testing, was lower when used to test specimens from asymptomatic (35.8%) than from symptomatic (64.2%) persons, but specificity was high. Sensitivity was higher for culture-positive specimens (92.6% and 78.6% for those from symptomatic and asymptomatic persons, respectively); however, some antigen test-negative specimens had culturable virus.

**What are the implications for public health practice?**

The high specificity and rapid BinaxNOW antigen test turnaround time facilitate earlier isolation of infectious persons. Antigen tests can be an important tool in an overall community testing strategy to reduce transmission.

## Footnotes

* Specimens were used to perform a limiting-dilution inoculation of Vero CCL-81 cells, and cultures showing evidence of cytopathic effect were tested by real-time RT-PCR for the presence of SARS-CoV-2 RNA. Viral recovery was defined as any culture in which the first passage had an N1 Ct value at least two Ct values lower than the corresponding clinical specimen.

† https://www.biorxiv.org/content/10.1101/2020.03.02.972935v1.

§ 45 C.F.R. part 46.102(l)(2), 21 C.F.R. part 56; 42 U.S.C. Sect. 241(d); 5 U.S.C. Sect. 552a; 44 U.S.C. Sect. 3501 et seq.

¶ Participants were asked whether they had each sign or symptom from a list based on Council for State and Territorial Epidemiologists clinical criteria for COVID-19 that included fever, cough, shortness of breath, fatigue, sore throat, headache, muscle aches, chills, nasal congestion, difficulty breathing, diarrhea, nausea, vomiting, abdominal pain, rigors, loss of taste, and loss of smell. https://cdn.ymaws.com/www.cste.org/resource/resmgr/ps/positionstatement2020/Interim-20-ID-02_COVID-19.pdf.

** Ct values from the N1 viral nucleocapsid protein gene region from real-time RT-PCR were compared only for specimens that were analyzed with the CDC 2019-nCoV Real-Time RT-PCR Diagnostic Panel for detection of SARS-CoV-2. Lower Ct values represent higher levels of viral RNA in the specimen and higher Ct values represent lower levels of viral RNA.

†† https://www.cdc.gov/coronavirus/2019-ncov/lab/faqs.html#Interpreting-Results-of-Diagnostic-Tests.

§§ https://www.cdc.gov/coronavirus/2019-ncov/prevent-getting-sick/prevention.html.
