# *Erratum:* Vol. 70, No. 3

**DOI:** 10.15585/mmwr.mm7004a6

**Published:** 2021-01-29

In the report “Evaluation of Abbott BinaxNOW Rapid Antigen Test for SARS-CoV-2 Infection at Two Community-Based Testing Sites — Pima County, Arizona, November 3–17, 2020,” on page 103, in Table 2, the positive predictive value (PPV) and 95% confidence interval (95% CI) for the BinaxNOW antigen test performance for asymptomatic participants should have read “**91.7 (80.0–97.7).**” Also on that page, the second sentence of the fourth full paragraph should have read “Community testing strategies focused on preventing transmission using antigen testing should consider serial testing (e.g., in kindergarten through grade 12 schools, institutions of higher education, or congregate housing settings), which might improve test sensitivity in **detecting** infection (*10*).”

